# Abalone visceral extract inhibit tumor growth and metastasis by modulating Cox-2 levels and CD8+ T cell activity

**DOI:** 10.1186/1472-6882-10-60

**Published:** 2010-10-20

**Authors:** Choong-Gu Lee, Ho-Keun Kwon, Jae Ha Ryu, Sung Jin Kang, Chang-Rok Im, Jae II Kim, Sin-Hyeog Im

**Affiliations:** 1 School of Life Sciences and Immune Synapse Research Center, Gwangju Institute of Science and Technology (GIST), Gwangju 500-712, Republic of Korea; 2 Global leader program, Bugil Academy, Cheonan, Chungchengnamdo 330-941, Republic of Korea

## Abstract

**Background:**

Abalone has long been used as a valuable food source in East Asian countries. Although the nutritional importance of abalone has been reported through *in vitro *and *in vivo *studies, there is little evidence about the potential anti-tumor effects of abalone visceral extract. The aim of the present study is to examine anti-tumor efficacy of abalone visceral extract and to elucidate its working mechanism.

**Methods:**

In the present study, we used breast cancer model using BALB/c mouse-derived 4T1 mammary carcinoma and investigated the effect of abalone visceral extract on tumor development. Inhibitory effect against tumor metastasis was assessed by histopathology of lungs. Cox-2 productions by primary and secondary tumor were measured by real-time RT-PCR and immunoblotting (IB). Proliferation assay based on [^3^H]-thymidine incorporation and measurement of cytokines and effector molecules by RT-PCR were used to confirm tumor suppression efficacy of abalone visceral extract by modulating cytolytic CD8+ T cells. The cytotoxicity of CD8^+ ^T cell was compared by JAM test.

**Results:**

Oral administration of abalone visceral extract reduced tumor growth (tumor volume and weight) and showed reduced metastasis as confirmed by decreased level of splenomegaly (spleen size and weight) and histological analysis of the lung metastasis (gross analysis and histological staining). Reduced expression of Cox-2 (mRNA and protein) from primary tumor and metastasized lung was also detected. In addition, treatment of abalone visceral extract increased anti-tumor activities of CD8^+ ^T cells by increasing the proliferation capacity and their cytolytic activity.

**Conclusions:**

Our results suggest that abalone visceral extract has anti-tumor effects by suppressing tumor growth and lung metastasis through decreasing Cox-2 expression level as well as promoting proliferation and cytolytic function of CD8^+ ^T cells.

## Background

Abalones are medium to very large-sized edible sea snails, marine gastropod mollusks in the family *Haliotidae *and the genus *Haliotis *[1]. Abalones are largely cultivated and used as valuable food resources in East Asian countries. In Korea, not only the protein-rich body part of abalone but also the viscera portion is taken in the form of sashimi or pickle and used as stamina food from ancient times. Although the nutritional composition of abalone visceral extract have not yet been identified in depth, since abalone live on brown algae like *Ecklonia*, *Laminaria *and *Undaria *for cultivation [2], it is regarded that the visceral portion of abalone may contain concentrated nutritional components derived from sea weed. Besides, it is well known that polysaccharides and glycoproteins of the brown algae possess potential immune-stimulant, anti-tumoral and anti-viral activity [3]. However, there is still not much information about the nutritional effects of abalone viscera itself.

There are some previous reports that the body and visceral portion of *Haliotis discus hannai *showed *in vitro *antioxidant activity [4,5]. Antioxidants are present in abundance in the dietary substances and their potent chemopreventive effects against many type of cancers have been reported [6]. The water extract of abalone has anti-tumor effects [7]. However, mechanisms involved in the anti-tumor effects of extract from abalone viscera have not been fully clarified. The purpose of this study is to evaluate the anti-tumor activity of abalone visceral extract *in vivo *using mouse breast cancer as a model and to elucidate underlying mechanisms involved in this protective effect.

Breast cancer is one of the major type of cancer affecting women and ranks second in causing death in women [8]. Although many clinical trials and basic research have been performed to subdue this disease, there is no conclusive treatment or medicine. Moreover, current conventional chemotherapies have severe side effects and toxicity [9,10]. Hence, there are increasing numbers of studies to prevent cancers using dietary factors [11,12]. To evaluate the anti-tumor effects of abalone visceral extract on breast cancer model, we used 4T1 murine mammary carcinoma cells that have highly metastatic characteristics at an early stage and mimic the human breast cancer [13,14]. Oral administration of abalone visceral extract significantly inhibited tumor progression by decreasing the levels of Cox-2, EGF, VEGF, and FGF in primary tumor as well as metastatic lesions. In addition, abalone visceral extract potentiated proliferation and cytolytic activity of CD8^+ ^T cells.

## Methods

### Animals

Balb/c mice were purchased from SLC (Hamamatsu, Japan) and maintained under specific pathogen-free conditions in the animal facility of the Gwangju Institute of Science and Technology. Animal experiments were performed in accordance with protocols approved by the animal care and use committees of the Gwangju Institute of Science and Technology.

### Preparation of the abalone visceral extract

Visceral portion of *Haliotis discus hannai *were obtained from live abalone. For 500 g of abalone visceral mass, we added 2 liters of 30% acetic acid then homogenized thoroughly. Then, the mixture was incubated at 4°C for 12 hours with rotation. Next, the mixture was centrifuged at 4°C for 1 hour with 5,000 rpm. Supernatant fraction was harvested and completely freeze-dried for 72 hours by using a freeze-dryer (Ilshin, Korea). The dried powder extract was further finely grounded by mortar and pestle and solubilized in sterilized PBS at 50 mg/ml concentration. Reverse phase high-performance liquid chromatography (RP-HPLC) assay was performed and relative amounts laminarin and D-mannitol were used as standard makers for the quality control of abalone visceral extract composition in each experiment (Additional file 1: Figure S1).

### Tumor induction and anti-tumor assay

Induction of breast cancer was carried out as described [15] with minor modifications. Mice were divided into two groups of abalone visceral extract-fed versus control PBS-fed. Sex- and age-matched mice with similar body weight were fed with either abalone visceral extract (5 mg/dose) or control PBS every day for 2 weeks prior to tumor transplantation. 1 × 10^6 ^cells/0.1 ml of 4T1 mouse mammary carcinoma cells were injected into a subcutaneous space on the flank of each mouse (female Balb/c mice from 6 to 8 weeks). Tumor size was measured with a Vernier caliper every 2 days from post-tumor induction, and tumor volumes were measured by the standard formula: width^2 ^× length × 0.52. Mice were sacrificed for further analysis at the 25 days from post-tumor induction.

### Cell isolation and culture

CD8^+ ^T cells were purified from the draining lymph nodes using CD8^+ ^T cell isolation kit (Miltenyi Biotech, Germany). For the activation, CD8^+ ^T cells were stimulated with 10 μg/ml plate-bound anti-CD3, 10 μg/ml soluble anti-CD28 in RPMI 1640 medium (Welgene, Korea) supplemented with 10% fetal bovine serum, L-glutamine, penicillin-streptomycin, nonessential amino acids, sodium pyruvate, vitamins, HEPES and β-mercaptoethanol. Anti-CD3 (145.2C11) and anti-CD28 (37.51) were purchased from BD Biosciences.

### Histopathology

Lung tissues of mice in each group were dissected and fixed in 4% formaldehyde. After embedding in paraffin, the tissue sections (5 μm) were mounted on glass slides and stained with hematoxylin and eosin following the previous report [15]. The specimens were observed under a light microscope (Olympus, Germany).

### RNA Isolation, cDNA synthesis, quantitative RT-PCR

Total RNA was extracted from the cells using TRIzol reagent (Molecular Research Center, USA) according to the manufacturer's protocol. For reverse transcription, cDNA was generated using 1 μg of total RNA, oligo(dT) primer (Promega) and Improm-II Reverse Transcriptase (Promega) in a total volume of 20 μl. One microliter of cDNA was amplified using the specific primers (Table. 1). Mouse hypoxanthine-guanine phosphoribosyl transferase (HPRT) primer was used for quantitative RT-PCR to normalize the amount of cDNA used for each condition.

**Table 1:** Primers used for RT-PCR

Genes	Sense	Antisense
HPRT	TTATGGACAGGACTGAAAGAC	GCTTTAATGTAATCCAGCAGGT
Cox-2	TGAGCAACTATTCCAAACCAGC	GCACGTAGTCTTCGATCACTATC
VEGF	GCACATAGAGAGAATGAGCTTCC	CTCCGCTCTGAACAAGGCT
FGF	ACCCACACGTCAAACTACAAC	CACTCCCTTGATAGACACAACTC
EGF	TTCTCACAAGGAAAGAGCATCTC	CTGCTGTCCCGTTAAGGAAAAC
MMP13	CTTCTTCTTGTTGAGCTGGACTC	CTGTGGAGGTCACTGTAGACT
GzmB	CCACTCTCGACCCTACATGG	GGCCCCCAAAGTGACATTTATT
GzmC	GCAGAGGAGATAATCGGAGGC	GCACGAATTTGTCTCGAACCA
FasL	TCCGTGAGTTCACCAACCAAA	GGGGGTTCCCTGTTAAATGGG
TNF-α	CATCTTCTCAAAATTCGAGTGACAA	TGGGAGTAGACAAGGTACAACCC
IFN-γ	GAGCCAGATTATCTCTTTCTACC	GTTGTTGACCTCAAACTTGG

### Proliferation assay

Assessment of lymphocyte responses against anti-CD3 and anti-CD28 stimulation was determined by [^3^H]-thymidine incorporation assays. Briefly, isolated CD8^+ ^lymphocytes were plated in flat-bottom 96-well plates and cultured in the presence or absence of stimulation. After 56 hours of incubation, 0.5 μCi of [^3^H]-thymidine (NEN, USA) was added, and cells were pulse labeled for 16 h. The degree of proliferation was presented as counts per minutes detected by scintillation counter (Beckman, USA).

### Total lysate preparation and immunoblotting

For total lysate preparation, tumor and lung tissues were first homogenized using homogenizer (Pro Scientific, USA) and then centrifuged briefly. Next, pelleted cells were lysed in RIPA buffer (50 mM Tris, pH 7.6, 150 mM NaCl, 1% NP-40) containing protease inhibitor cocktails (Roche, USA) for 10 min on ice. To determine levels of Cox-2 proteins, total lysates from each sample were prepared described above and loaded together with a PageRuler Prestained Protein ladder (Fermentas, Canada) on a 10% SDS-PAGE gel. The proteins were electroblotted onto a nitrocellulose membrane (Bio-Rad, USA). After blocking, the membranes were incubated with a 1/500 dilution of anti-Cox-2 (Cayman chemical, USA) in blocking buffer overnight at 4°C. The blots were developed using a 1/10,000 diluted anti-rabbit HRP (Abcam, USA) and visualized by ECL solution (Amersham, UK). To confirm the amount of sample loading and transfer, membranes were incubated in stripping buffer, re-blocked for 1 h, and reprobed with anti-β-tubulin (Santa Cruz) with anti-mouse HRP (Abcam).

### JAM test

The cytotoxicity of CD8^+ ^T cell was compared by JAM test with minor modification [16]. EL4 mouse lymphoma cells were used as target cells. Target cells were labeled with 5 μCi/ml of [^3^H]-thymidine (NEN, USA) for 12 hours at 37°C. Labeled target cells were harvested, washed 3 times with PBS, and seeded in 96-well flat-buttom plates at a density of 1 × 10^4 ^cells/well. CD8^+ ^T cells were used as effector cells. Activated CD8^+ ^T cells were washed and added in various ratios to the target cells. After 16 hours co-culture, remained radioactivity from intact cells can be trapped in the filter paper and then the filter discs were measured on a microplate beta counter (Beckman, USA). Data were expressed as the percentage of cytotoxicity calculated by the following formula: [(cpm spontaneous - cpm experiment)/cpm spontaneous] × 100.

### Statistical analysis

All the data were expressed as mean ± standard error (SE). For statistical analysis, two-tailed student's *t*-test was employed unless otherwise stated. Differences were considered statistically significant with a *p*-value of ≤ 0.05 (* < 0.05, ** < 0.01, *** < 0.001).

## Results

### Administration of abalone visceral extract suppresses tumor growth

To ensure the quality and purity of each preparation of abalone visceral extract, RP-HPLC analysis was performed by measuring the content of known active compounds such as laminarin and D-mannitol which are known as major polysaccharide constituents in *Laminaria*, the food for abalone. (Additional file 1: Figure S1).

**Figure 1:**
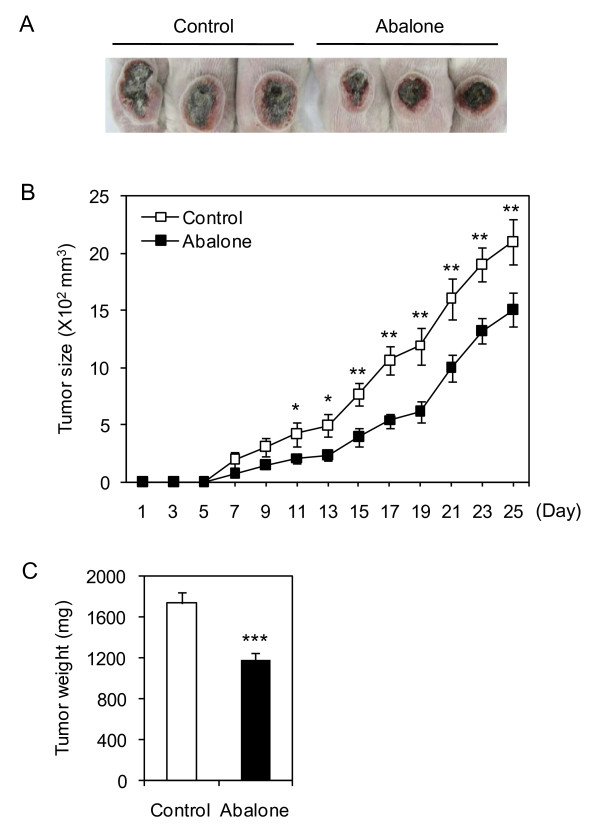
**Suppression of tumor progression by administration of abalone visceral extract**. Balb/c female mice were orally administered with abalone visceral extract (100 ul of 50 mg/ml) for 2 weeks before cancer transplantation. Mouse breast cancer model was induced by injection of 4T1 cell (1 × 10^6 ^cells) into flank backs. Abalone visceral extract or PBS as control was daily treated by oral administration for 25 days post tumor induction. (A) Picture of tumor from each group was taken. (B) During the treatment period, tumor size was measured every 2 days and shown in graph. (C) After 25 days from post-tumor induction, mice from each group were scarified and the weight of tumor mass was measured. Error bars indicated SE. Data are representative of five independent experiments. One (*), two (**) or three asterisks (***) indicate p < 0.05, p < 0.01 or p < 0.001, respectively.

To determine the dose of abalone visceral extract that does not induce *in vivo *toxicity upon oral administration, Balb/C mice were fed with 5 mg of abalone visceral extract using feeding needle every other day for 20 days and the body weight was measured (Additional file 2: Figure S2A). Overall, mice viability and changes in body weight were not affected by administration of abalone visceral extract. In addition, gross analysis of the intestine did not show any signs of inflammation both in the control (PBS) and abalone visceral extract fed group (Additional file 2: Figure S2B). Anti-cancer effect of abalone visceral extract was tested in mouse breast cancer model in Balb/C as described in Material and Method section. Mice were fed either with 5 mg of the abalone visceral extract or PBS as a control for 2 weeks. And then breast cancer was induced by intradermal injection of 4T1 mouse mammary carcinoma cells. Oral administration of abalone visceral extract continued for additional 25 days till the end of experiment. Administration of abalone visceral extract significantly decreased the size of tumor formation compared with control groups in overall. Administration of abalone visceral extract significantly (about 30%) reduced tumor size compared with control group by gross analysis (Figure. 1A). During the treatment periods, tumor size was measured starting from day 7 of post-induction of cancer and till the end of experiments, 25 day of post-induction (Figure. 1B). In addition, administration of abalone visceral extract significantly reduced tumor mass compared with the control group (Figure. 1C). These data suggest that oral treatment of abalone visceral extract significantly reduced the development and progress of tumor formation in breast cancer model.

### Administration of abalone visceral extract inhibits tumor metastasis

**Figure 2:**
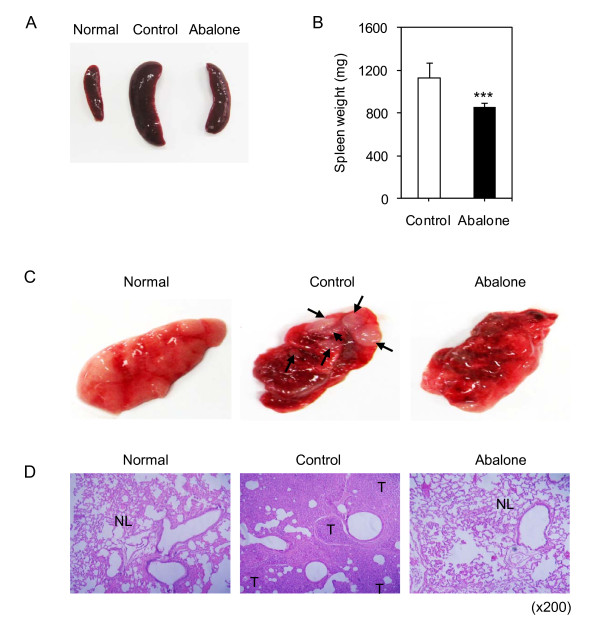
**Effects of abalone visceral extract on tumor metastasis**. (A) The gross analysis of spleen from normal healthy, fed with abalone visceral extract or PBS control treated group. (B) Spleen weight was measured and shown in graph. Values are means with SE, n = 5. Data are representative of five independent experiments. One (*), two (**) or three asterisks (***) indicate p < 0.05, p < 0.01 or p < 0.001, respectively. (C) Gross appearance of lung from each treatment was shown and compared with normal healthy lung. Arrows from control lung picture indicate metastatic foci (middle panel). (D) H&E-stained section of lung from each group was shown. Normal lung tissue is indicated by 'NL'. Tumor metastases are indicated by 'T'. Original magnifications; ×200

The 4T1 breast cancer cells are one of the highly metastatic cancer cells [14] and metastatic splenomegaly is known as the first manifestation of metastasis in breast cancer [17,18]. Oral administration of the abalone visceral extract reduced the lymphomegaly (data not shown) and metastatic splenomegaly as evident from the decreased size (Figure. 2A) and weight of spleen (Figure. 2B). From these results, we hypothesized that abalone visceral extract could suppress the metastasis of the primary tumor. Since lung is the one of the predominant site of metastasis in breast cancer [19-21], we first compared the metastatic state of lungs between the experimental groups by gross examination (Figure. 2C). Control group showed deposited secondary tumors with various sizes (Figure. 2C, middle panel). In contrast, the lung of abalone visceral extract fed group showed no secondary tumors (Figure. 2C, right panel). For more detailed examination of metastatic state of lungs from each group, histological examination was performed by hematoxylin and eosin (H&E) staining (Figure. 2D). Unlikely the normal alveolar structure of normal healthy lungs (Figure. 2D, left panel), the control group showed massive infiltration of the tumors with increased numbers of small lymphatic or blood vessels (Figure. 2D, middle panel). Interestingly, lung from the abalone visceral extract treated group showed normal alveolar structure similar to the normal healthy group while control group showed destructed alveolar (Figure. 2D, right panel). These data indicate that treatment of abalone visceral extract inhibited metastatic development of breast cancer in lung tissues.

### Administration of abalone visceral extract suppress primary tumor growth by decreasing Cox-2 expression

**Figure 3:**
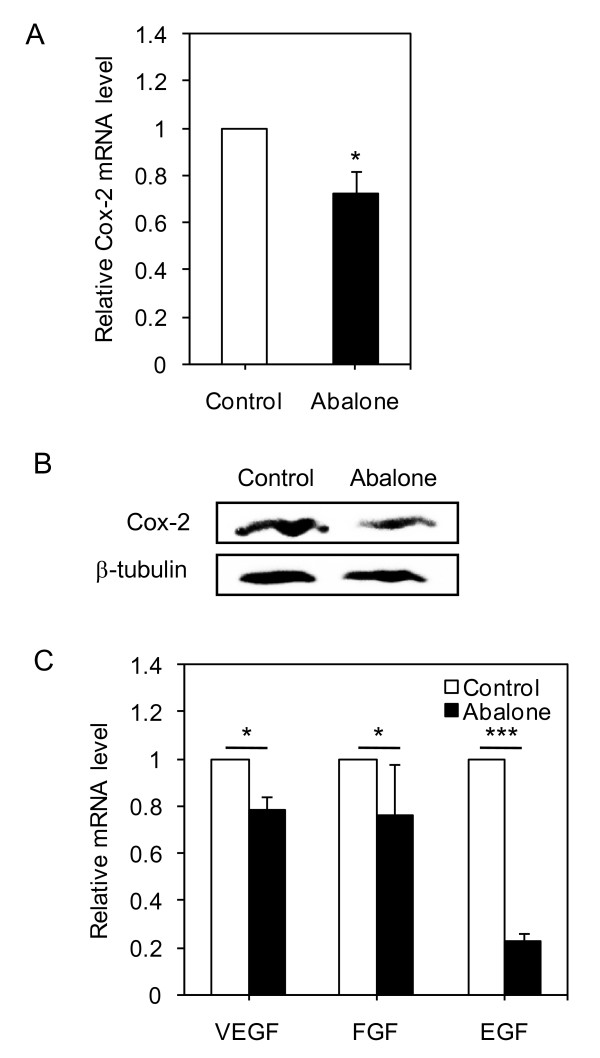
**Effect of administration of abalone visceral extract on the levels of Cox-2 and angiogenic markers in primary tumor**. (A) Primary tumor mass were homogenized and Cox-2 mRNA level was measured in each group. (B) Protein level of Cox-2 was detected using total cell lysates from each group. β-tubulin was used as loading control. (C) Angiogenic markers of tumor growth were measured by quantitative real-time PCR. Values are means with SE. Data are representative of five times independent experiments. One (*), two (**) or three asterisks (***) indicate p < 0.05, p < 0.01 or p < 0.001, respectively.

Elevated Cox-2 expression is associated with increased tumor size during breast cancer progression [22], while specific knockdown of Cox-2 directly reduced level of PGE_2 _synthesis and tumor cell growth in 4T1 cells [23]. We investigated whether anti-cancer effect of abalone visceral extract (Figure. 1 and Figure. 2) is linked with modulation of Cox-2 expression level. Since Cox-2 is regulated at transcriptional and post-translational levels [24], Cox-2 transcript and protein levels were analyzed by real-time PCR and Western blot analysis, respectively from the tumor cells of breast cancer mice treated either with control (PBS) or abalone visceral extract. Mice fed with abalone visceral extract showed significantly reduced Cox-2 mRNA (Figure. 3A) and protein (Figure. 3B) levels compared to PBS fed control group. Tumorigenesis is often accompanied with angiogenic development [25]. Therefore, we further investigated whether oral administration of abalone visceral extract could also affect expression levels of VEGF, FGF and EGF. Indeed, abalone visceral extract treated group showed significantly reduced mRNA level of such targets molecules in the tumor tissues (Figure. 3C). These results suggest that the potent anti-tumor effect of abalone visceral extract is associated with down-regulation of Cox-2 expression level, as well as reduced transcript levels of tumor growth related angiogenic molecules such as VEGF, FGF and EGF in tumor tissues.

### Abalone visceral extract inhibits tumor metastasis by modulating Cox-2 expression

**Figure 4:**
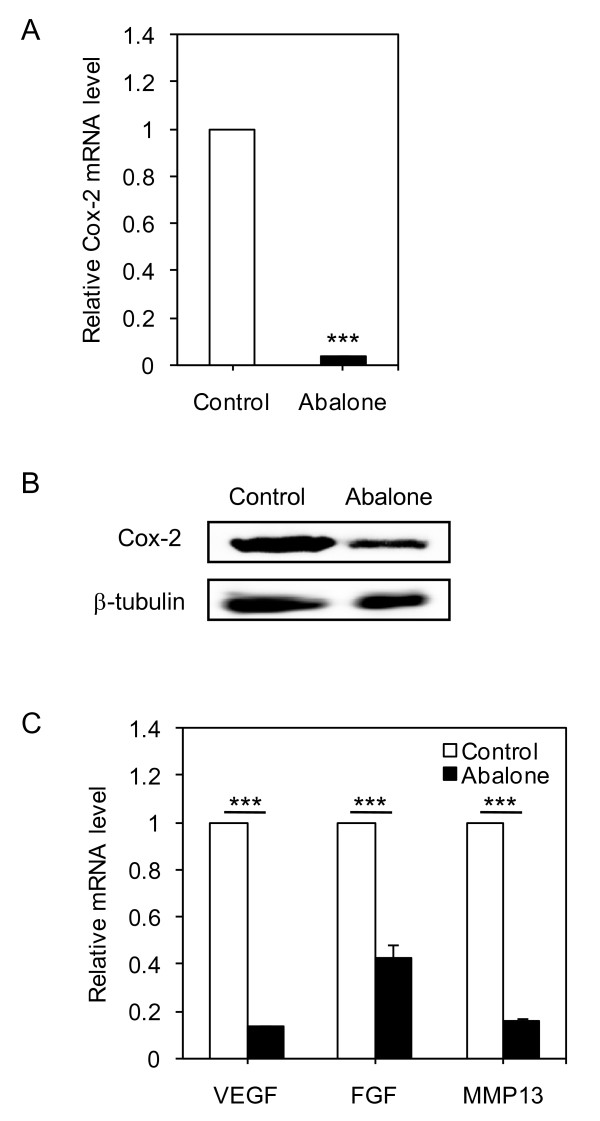
**Abalone visceral extract inhibits tumor metastasis by modulating Cox-2 and Cox-2 related tumor metastasis markers**. (A) Metastasized lung mass were homogenized and Cox-2 mRNA level was measured in each group by quantitative real-time PCR. (B) Protein level of Cox-2 was detected using total cell lysates from each group. β-tubulin was used as loading control. (C) Markers of tumor metastasis were measured by quantitative real-time PCR. Values are means with SE. Data are representative of five times independent experiments. One (*), two (**) or three asterisks (***) indicate p < 0.05, p < 0.01 or p < 0.001, respectively.

Cox2 plays important roles in breast cancer metastasis to bone [26] and increased Cox-2 level was identified as one of the markers for metastasis [27]. Therefore, we questioned whether suppression of tumor metastasis by oral administration of abalone visceral extract correlates with down-regulation of Cox-2 level in metastatic tissue. The mRNA and protein levels of Cox-2 were determined from the lung tissue isolated from each treatment group. Indeed, administration of abalone visceral extract significantly reduced Cox-2 levels compared to PBS fed group both in mRNA (Figure. 4A) and protein (Figure. 4B) levels. To further check whether oral administration of abalone visceral extract also affected expression levels of metastasis related molecules, we analyzed the mRNA levels of *VEGF*, *FGF *and *MMP-13 *[28,29]. Indeed, administration of abalone visceral extract significantly decreased their expression levels (Figure. 4C). Taken together, these data suggest that the potent anti-metastatic effect of abalone visceral extract is associated with down-regulation of Cox-2 expression in metastatic lesions, as well as suppression of metastatic targets molecules such as VEGF, FGF and MMP-13.

### Abalone visceral extract potentiates the CD8^+ ^cytotoxic T cell activity

**Figure 5:**
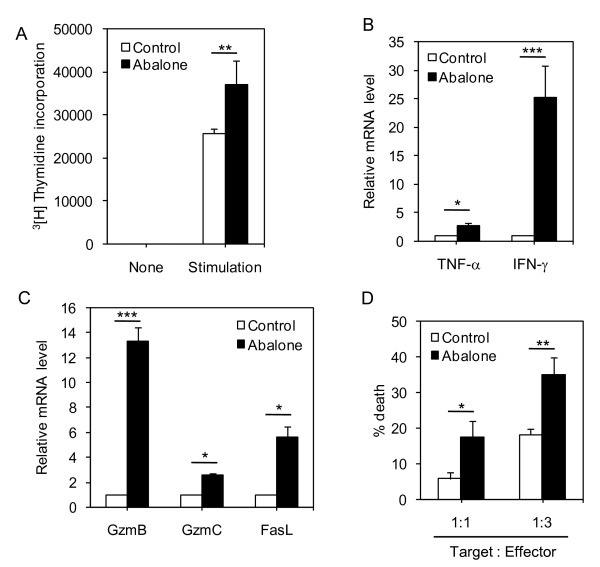
**Treatment of abalone visceral extract enhances the cytolytic activity of CD8^+ ^T cells**. After 25 days of abalone visceral extract treatment, CD8^+ ^T cells in tumor draining lymph nodes (axillary, superficial, brachial and linguinal lymph node) were isolated from each group. (A) Proliferative capacity of CD8^+ ^T cells from each group was tested by [^3^H]-thymidine incorporation assay. (B and C) The expression levels of cytokines (TNF-α, IFN-γ) and cytolytic molecules such as granzyme B (GzmB), granzyme C (Gzmc) and FasL) were measured by quantitative real-time PCR. The mRNA expression levels in each group were normalized with HPRT (house-keeping gene) and then fold induction of each target gene was compared to PBS groups (expression level of control group: 1). (D) The cytotoxicity of CD8^+ ^T cells was compared by JAM test. CD8^+ ^T cells were co-cultured with [^3^H]-thymidine labeled EL4 T cells for 12 hours and the radioactivity in each sample was measured. Values are means with SE. One (*), two (**) or three asterisks (***) indicate p < 0.05, p < 0.01 or p < 0.001, respectively.

Chemopreventive approaches for tumor cells are often targeting modulation of tumor environment not only by exerting anti-tumor activity but also by regulating the immune system [30]. Among various types of immune cells, CD8^+ ^cytotoxic T cells are well known for their anti-tumor activity by directly killing transformed cells through the perforin-granzyme pathway and death ligand such as Fas and tumor-necrosis factor [31]. We tested the possibility that the anti-tumor activity of abalone visceral extract may also modulate the functional activity of CD8^+ ^T cells. Firstly, we performed CD8^+ ^T cell proliferation assay. Abalone visceral extract-fed group showed significantly increased (~30%) proliferation capacity compared with control CD8^+ ^T cells from PBS-fed group (Figure. 5A). And then, the expression levels of cytokines related with effector function of CD8^+ ^T cells were determined by quantitative RT-PCR. TNF-α and IFN-γ are known important mediators of functionality of CD8^+ ^T cells against tumor pathogenesis [32]. CD8^+ ^T cells from the abalone visceral extract-fed groups showed significantly increased levels of IFN-γ and TNF-α (Figure. 5B). Then, we compared the mRNA levels of genes related with cytolytic activity of CD8^+ ^T cells such as granzymes (gzmB and gzmC) and Fas ligand (FasL) (Figure. 5C). CD8^+ ^T cells from the extract-fed group showed increased mRNA level of the above target genes compared with CD8^+ ^T cells from the control group. Since the expression level of cytolytic molecules is directly linked with the killing activity of CD8^+ ^T cells [33], we tested the hypothesis that CD8^+ ^T cells from the abalone visceral extract-treated groups exhibited an increased CTL response than those from the control group. We performed the JAM test to test this hypothesis. Indeed, CD8^+ ^T cells from the abalone visceral extract-treated groups showed significantly increased killing activity than that of the control group in a dose-dependent manner (Figure. 5D). These results indicate that abalone visceral extract potentiates the proliferative capacity and cytolytic activity of CD8^+ ^T cells in tumor draining lymph nodes.

## Discussion

Abalone is often called as 'The emperor of the seashells' in Korea and mostly consumed as stamina food for sick and weak individuals because of its high content of proteins and vitamins [5]. Abalone mainly live on sea weeds that contain rich and concentrated nutritional components [2]. Apart from its known nutritional importance, there are not many studies on the effects of the abalone visceral extract. One of the known effects of abalone visceral extract is its antioxidant activity as demonstrated [4,5]. However, there are still few *in vivo *evidence and no detailed action mechanisms for its anti-tumor effects [7]. In the present study, we have demonstrated the potent anti-tumor efficacy of abalone visceral extract and have elucidated its underlying mechanism using a mouse breast cancer model that have high malignancy in tumor growth and metastasis [13,14]. Oral administration of abalone visceral extract significantly lowered tumor progression and metastasis by down-regulating the tumor-associated growth factors such as Cox-2, EGF, VEGF and FGF, while increasing the proliferation and cytolytic activity of CD8^+ ^T cells.

Cyclooxygenase-2 (Cox-2) is an enzyme that catalyzes arachidonic acid to prostaglandins. Cox-2 is predominantly expressed in synoviocytes, fibroblasts, osteoblasts, activated endothelial cells and tumor cells [34,35]. Cox-2 expression is induced by pro-inflammatory and mitogenic stimuli such as growth factors (EGF, FGF and VEGF) [36] and cytokines (TNF-α and IL1-β). Enhanced expression of Cox-2 is linked with tumor progression by inducing immune suppression as well as angiogenic and metastatic progression [34,37,38]. Elevated Cox-2 expression is associated with increased tumor size during breast cancer progression [22,39]. Modulation of Cox-2 expression by specific inhibitors is regarded as good chemopreventive approach for cancer treatment. However, Cox-2 inhibitors affect multiple cellular pathways and show some side effects [34,40]. Therefore, use of nutritive supplementation substances might be regarded as potential cancer preventive approach [11,12]. We have found that the oral administration of abalone visceral extract exerted anti-tumor growth effects by inhibiting tumor volume (up to 30%) compared with control feeding group (Figure. 1). The mouse breast tumor induced by 4T1 tumor cells mimics human breast cancer in the aspect of spontaneous metastasis to lung, lymph nodes, liver and bone [41]. Cox-2 expression has been identified as the marker for selective lung metastasis [27] in breast cancer model [20]. In this study, we used 4T1 mammary adenocarcinoma cells for tumor implantation. Oral administration of abalone visceral extract significantly inhibited tumor metastasis by modulating Cox-2 expression (Figure. 4). In accordance with our result, a previous study also demonstrated that treatment of either non-selective Cox inhibitor or selective Cox-2 inhibitor significantly reduced primary tumor growth and metastasis of 4T1 breast cancer cells[42]. Even though reduction of Cox-2 expression does not exactly match with inhibition of Cox-2 activity by known inhibitors, specific knockdown of Cox-2 directly could reduce level of PGE2 synthesis in 4T1 cells [23]. In line with aforementioned reports, we investigated whether abalone visceral extract changes Cox-2 expression upon treatment. Oral administration of abalone visceral extract reduced the metastatic splenomegaly (Figure. 2A and 2B) [17,18] and lymphomegaly (data not shown) [43]. Metastatic breast cancer has a strong tendency to propagate into lung and bone [19-21,26]. Treatment of abalone visceral extract significantly inhibited lung metastasis (Figure. 2C and 2D) by decreasing Cox-2 expression level (Figure. 4A and 4B). Numerous evidences show that decreased level of Cox-2 is well correlated with metastatic inhibition from variety kinds of cancers [44]. Furthermore, previous data suggested that Cox-2 expression is associated with angiogenesis, lymph node metastasis, and apoptosis in human breast cancer [43] along with enhanced MMP-13 expression [28]. Interestingly, the expression levels of VEGF, EGF and MMP-13 are all decreased upon abalone visceral extract treatment (Figure. 4C). Collectively, oral administration of abalone visceral extract reduced metastatic progression by lowering Cox-2 expression and other target molecules including angiogenic factors and metalloproteinases in the metastatic tissues.

The tumor microenvironment induces active immune tolerance and escapes immune surveillance. Boosting the immune response can be one of the indirect ways to eliminate or suppress tumor growth via regulating immune homeostasis [30]. CD8^+ ^T cells are known to have anti-tumor activity by killing the tumor antigens in an antigen specific or antigen non-specific way [44,45]. Tumor specific CD8^+ ^T cells possess increased proliferation, cytolytic activity and induce expression of death related proteins and cytokines [31]. However, CD8^+ ^T cells at tumor sites or tumor draining lymph nodes frequently exhibit functional defects such as defective antigen specific cytolytic activity [46], lack of perforin expression [47], defective cytokine production and abnormal proliferation [48,49]. Enhanced CD8^+ ^T cell activity is therefore critical to eradicate tumor cells, especially in tumor regions. In this study, oral administration of abalone visceral extract significantly inhibited tumor growth compared with the control (PBS-treated) group (Figure. 1 and Figure. 2). Administration of abalone visceral extract enhanced the cytolytic activity of CD8^+ ^T cells by increasing the expression of effector molecules such as cytokines (IFN-γ and TNF-α) and cytolytic molecules (FasL, Gzm B and Gzm C) (Figure. 5B and 5C). Even though inflammatory cytokine signaling is the known stimulation for Cox-2 expression [24], increased expression of the cytokine in CD8+ T cells upon abalone visceral extract treatment can be explained by other mechanisms apart from Cox-2 regulation by abalone visceral extract in tumor cells. In addition, abalone visceral extract significantly increased the specific lysis rate in the JAM test (Figure. 5D). Therefore, the enhanced effector function of CD8^+ ^T cells by administration of abalone visceral extract may enhance anti-tumor immunity, which leads to suppression of tumor growth and metastasis to different organs.

## Conclusions

Our data suggest that abalone visceral extract suppress primary tumor formation and inhibit tumor metastasis by attenuating the expression of Cox-2 and other target molecules including angiogenic factors and metalloproteinases. Furthermore, abalone visceral extract potentiate immune responses of CD8^+ ^T cells by increasing their proliferation and cytolytic activity. Although further studies are needed to elucidate the exact active compounds responsible for the anti-tumor activity of abalone visceral extract, our data suggest the potential use of abalone visceral extract as an inhibitor of tumor growth and metastasis by targeting Cox-2 activity and the cytolytic effector function of CD8^+ ^T cells.

## List of abbreviations used

COX-2: Cyclooxygenase-2; VEGF: Vascular endothelial growth factor; FGF: Fibroblast growth factor; EGF: Epidermal growth factor; MMP13: Matrix metallopeptidase 13; GZMB: Granzyme B; GZMC: Granzyme C; FASL: Fas Ligand; TNF-α: Tumor necrosis factor-alpha; IFN-γ: Interferon-gamma.

## Competing interests

The authors declare that they have no competing interests.

## Authors' contributions

S-H.I and J.I.K designed the research; C-G.L, H-K.K and C-R.I conducted research; J.H.R and S.J.K helped the HPLC experiments and analysis; C-G.L and S-H.I analyzed data; C-G.L and S-H.I wrote the paper; S-H.I had primary responsibility for final content. All authors read and approved the final manuscript.

## Pre-publication history

The pre-publication history for this paper can be accessed here:

http://www.biomedcentral.com/1472-6882/10/60/prepub

## Supplementary Material

Additional file 1 **Supplementary Figure 1**. Quantitative analysis of Laminarin and D-mannitol in abalone visceral extract using reverse phase high-performance liquid chromatography (RP-HPLC). Click here for file

Additional file 2 **Supplementary Figure 2**. Toxicity test of the abalone visceral extract upon oral administration. Click here for file
