# Supplementary material for: Abalone visceral extract inhibit tumor growth and metastasis by modulating Cox-2 levels and CD8+ T cell activity
Source: BMC Complement Altern Med. 2010 Oct 20;10:60. doi: 10.1186/1472-6882-10-60 (PMC2972231; doi:10.1186/1472-6882-10-60)
Supplement: Additional file 1 — Supplementary Figure 1. Quantitative analysis of Laminarin and D-mannitol in abalone visceral extract using reverse phase high-performance liquid chromatography (RP-HPLC). [file 1472-6882-10-60-S1.PDF]

Supplementary Fig. 1.

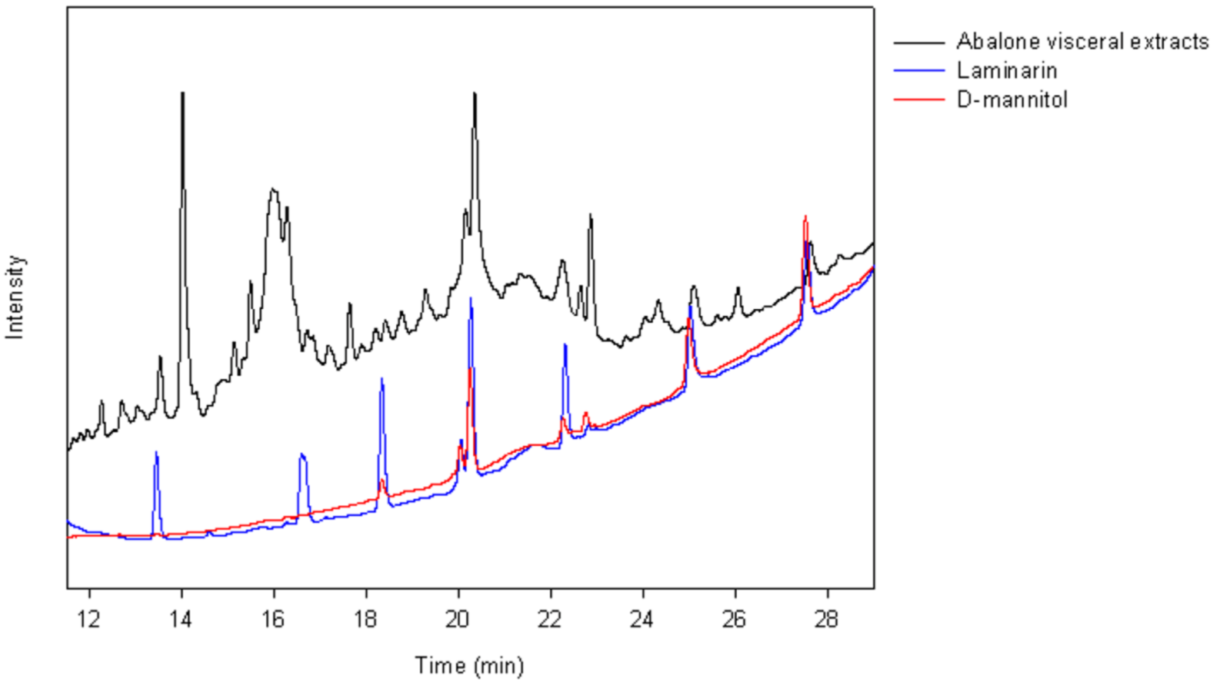

Supplementary Fig. 1. Quantitative analysis of Laminarin and D-mannitol in abalone visceral extracts using reverse phase high-performance liquid chromatography (RP-HPLC). RP-HPLC analysis was performed by comparing the levels of laminarin (Sigma, USA) and D-mannitol (Sigma, USA) known as major polysaccharide constituents in *Laminaria*, the food for abalone. Chromatography was carried out using H_2O and ACN with 5%-65% gradient at room temperature on a C18 column (4.6 x 250 mm). The flow rate of the mobile phase was 1ml/min. Detection wavelength at 230 nm.
