# Supplementary material for: Abalone visceral extract inhibit tumor growth and metastasis by modulating Cox-2 levels and CD8+ T cell activity
Source: BMC Complement Altern Med. 2010 Oct 20;10:60. doi: 10.1186/1472-6882-10-60 (PMC2972231; doi:10.1186/1472-6882-10-60)
Supplement: Additional file 2 — Supplementary Figure 2. Toxicity test of the abalone visceral extract upon oral administration. [file 1472-6882-10-60-S2.PDF]

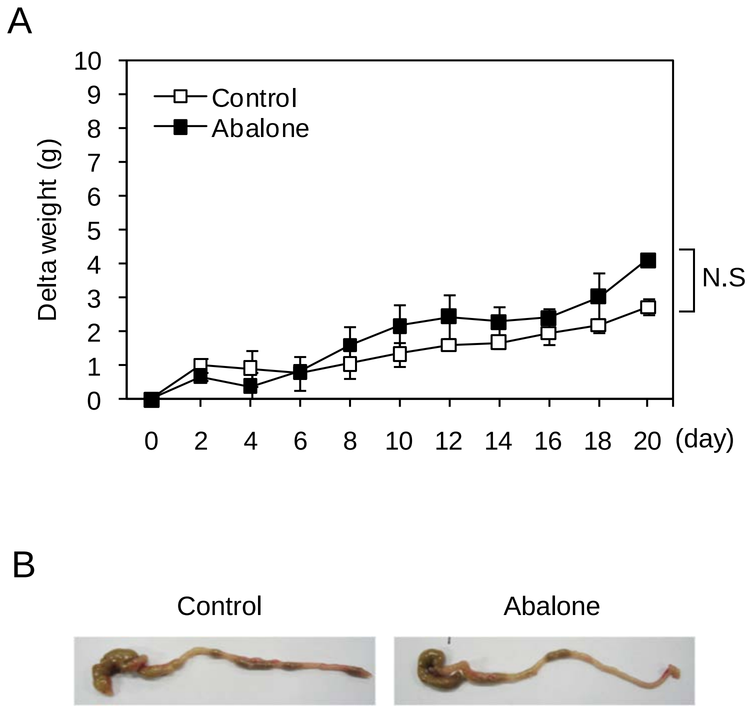

Supplementary Fig. 2. Toxicity test of the abalone visceral extracts upon oral administration. Mice were given 100ul of 50mg/ml of abalone visceral extracts by oral injection for 20 days. (A) Weight changes were measured every two days and shown as delta weight. N.S; statistically non-significant. (B) After 20 days from oral feeding, gross analysis of intestines from each group was performed.
